# Evaluation of a pig femoral head osteonecrosis model

**DOI:** 10.1186/1749-799X-5-15

**Published:** 2010-03-06

**Authors:** Ping Zhang, Yun Liang, Harry Kim, Hiroki Yokota

**Affiliations:** 1 Department of Biomedical Engineering, Indiana University - Purdue University Indianapolis, Indianapolis IN 46202, USA; 2 Department of Anatomy & Cell Biology, Indiana University School of Medicine, Indianapolis IN 46202, USA; 3 Department of Radiology, Indiana University - Purdue University Indianapolis, Indianapolis IN 46202, USA; 4 Shriners Hospital for Children, and University of South Florida, Tampa FL 33612, USA

## Abstract

**Background:**

A major cause of osteonecrosis of the femoral head is interruption of a blood supply to the proximal femur. In order to evaluate blood circulation and pathogenetic alterations, a pig femoral head osteonecrosis model was examined to address whether ligature of the femoral neck (vasculature deprivation) induces a reduction of blood circulation in the femoral head, and whether transphyseal vessels exist for communications between the epiphysis and the metaphysis. We also tested the hypothesis that the vessels surrounding the femoral neck and the ligamentum teres represent the primary source of blood flow to the femoral head.

**Methods:**

Avascular osteonecrosis of the femoral head was induced in Yorkshire pigs by transecting the ligamentum teres and placing two ligatures around the femoral neck. After heparinized saline infusion and microfil perfusion via the abdominal aorta, blood circulation in the femoral head was evaluated by optical and CT imaging.

**Results:**

An angiogram of the microfil casted sample allowed identification of the major blood vessels to the proximal femur including the iliac, common femoral, superficial femoral, deep femoral and circumflex arteries. Optical imaging in the femoral neck showed that a microfil stained vessel network was visible in control sections but less noticeable in necrotic sections. CT images showed a lack of microfil staining in the epiphysis. Furthermore, no transphyseal vessels were observed to link the epiphysis to the metaphysis.

**Conclusion:**

Optical and CT imaging analyses revealed that in this present pig model the ligatures around the femoral neck were the primary cause of induction of avascular osteonecrosis. Since the vessels surrounding the femoral neck are comprised of the branches of the medial and the lateral femoral circumflex vessels, together with the extracapsular arterial ring and the lateral epiphyseal arteries, augmentation of blood circulation in those arteries will improve pathogenetic alterations in the necrotic femoral head. Our pig model can be used for further femoral head osteonecrosis studies.

## Background

Osteonecrosis of the femoral head and neck is one of the major orthopedic diseases of bone degradation in the hip joint [1-4]. It can lead to collapse of the femoral head, resulting in permanent deformity and premature degenerative arthritis. Osteonecrosis usually affects individuals with a mean age in the 30's [5], but it can also affect children. Many etiologies such as trauma, radiation, exposure to corticosteroid use, alcohol intake, and various chronic diseases are considered to be associated with femoral head degeneration [6]. One of the primary pathomechanisms is, however, interruption of blood supply to the proximal femur.

Currently, various invasive and non-invasive treatments are used to prevent femoral head collapse [7]. Operative treatment options include core decompression, bone grafting, osteotomy and vascularized fibular grafting [8,9]. Despite those treatments, the femoral head tends to eventually deteriorate and collapse over time, leading to hip joint arthritis. Many such patients receive total hip arthroplasty in its late stage. Although total hip arthroplasty is an established surgical procedure, there are potentially serious risks involved with the procedure including deep venous thrombosis, pulmonary embolism, bone fracture during and after surgery, limitation of motion of the hip, and loosening of the prosthesis.

A number of non-operative treatments have been proposed and some of them have been clinically tested [10]. They include restricted weight-bearing [11], lipid-lowering agents [12,13], anticoagulants [14], vasodilators [15,16], bisphosphonates [17-19], shock-wave therapy [20], and application of pulsed electromagnetic fields [21,22]. The results of joint-preserving procedures are, however, less satisfactory than the results with total hip arthroplasty. Clinical studies support the notion that bisphosphonate can retard disease progression by suppressing osteoclastic activities. Further investigations are, however, needed to evaluate efficacy of bisphosphonate-based therapy for its long-term usage.

For development of a joint-preserving therapy, it is important to establish a suitable animal model and determine the primary cause of induction of avascular osteonecrosis of the femoral neck [23-25]. In the present study using an osteonecrosis model of Yorkshire pigs, we addressed the following questions: What arteries serve as major sources of blood circulation to the femoral head? Furthermore, are there transphyseal vessels that allow circulatory linkage between the epiphysis and the metaphysic [26,27]? Through transection of the ligamentum teres and ligature of the vessels surrounding the femoral neck, we examined the role of various vessel networks in blood circulation to the femoral head and induction of avascular osteonecrosis. To evaluate blood circulation in the proximal femur, a radiopaque agent (microfil) was perfused for optical and CT imaging.

## Methods

### Surgical Procedure to Induce Osteonecrosis

**Figure 1:**
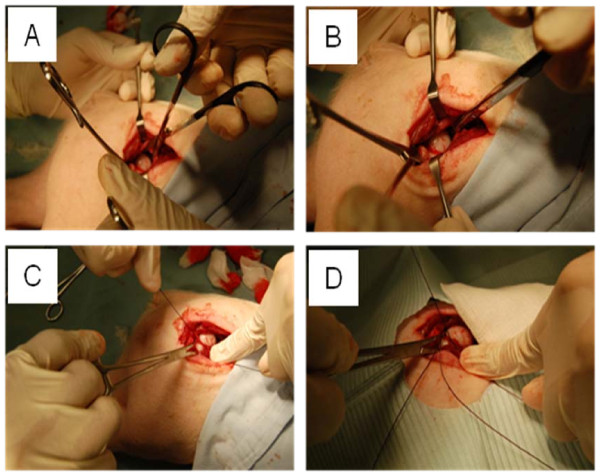
**Surgical procedure to induce avascular osteonecrosis of the pig femoral head**. (A) & (B) Ligamentum teres were visualized and surgically cut. (C) & (D) Two sutures were passed around the femoral neck and tied tightly to block a blood supply to the femoral head.

Use of female Yorkshire pigs (5 week old; 6 - 8 kg) was approved by the IACUC. Following pre-medication, general anesthesia was induced with 2% isoflurane. A longitudinal incision was made over the hip. Gluteus and hip abductor muscles overlying the hip joint were identified and separated using retractors. The hip joint capsule was partially incised to expose the lateral aspect of the femoral head and neck. The ligamentum teres were visualized by subluxing the femoral head and transecting with a curved scissor. Two sutures (#1 Vicryl, Ethicon) were then passed around the femoral neck and tied tightly to disrupt the blood vessels leading to the femoral head (Figure 1) [23,24]. To evaluate the contribution of the extracapsular arterial ring to the femoral head, the two ligatures to the control pig were sham operated (two sutures around the femoral neck were not tied). Surgical wounds were closed in multi-layers using #3-0 Vicryl (Ethicon) and #4-0 PDS II (Ethicon).

### Preparation of Microfil Casts

At 3 and 48 h post induction of ischemia, microfil was perfused for detection of blood circulation. Following pre-medication and general anesthesia, a midline incision was made to expose the abdominal aorta and the inferior vena cava. A needle (#18) was distally inserted, and the cannula was used to inject 60 ml of heparinized saline (10,000 units in 0.9% sodium chloride) with pressure at 100 mmHg via the abdominal aorta. A radiopaque, lead-containing liquid, low-viscosity polymer (Microfil MV-117 or MV-122, Flow Tech; Carver, MA) was then infused with an external pressure of 100 mmHg. The infusion volume was ~60 ml, and the agent flowed freely through the inferior vena cava. After perfusion, the sample was placed at 4°C overnight to allow polymerization of the polymer [28].

### CT Imaging

In order to evaluate blood circulation in the necrotic and control proximal femora, CT imaging was performed on the microfil casted samples. The three-dimensional geometries of the vascular systems together with femoral structures were reconstructed with a resolution of ~400 μm in a transverse direction and ~700 μm in a cranial caudal direction [29,30]. To evaluate the radiodensity of microfil casted samples, we determined image density in Hounsfield Units (HU, standard CT density unit). In the present study, a higher image density indicated greater blood perfusion (circulation) and bone density. Four particular regions of interest included a femoral head and neck, a whole femoral head, a proximal end of the femoral head, and a base of the femoral head.

### Optical Imaging of Vascular Anatomy

After CT imaging, the bone samples including the surrounding tissues were harvested using a surgical microscope (Series SSI-202/402, Seiler Instrument Microscope Division). Blood circulation to the proximal femur was traced from the vascular branches in the femoral artery system towards the femoral head and neck. A mid cross-section of the femoral head (~500 μm) was removed, and microfil casted blood vessels were imaged using a Nikon E4500 digital camera [31].

## Results

The animals used for induction of avascular osteonecrosis of the femoral head tolerated the procedures, and no abnormal behavior was observed.

### Vascular Anatomy

We first observed the gross arterial networks of the femoral head and neck using the microfil casted samples with surgical microscopy. Multiple branches stemmed from the medial femoral circumflex artery (MFCA) and the lateral femoral circumflex artery (LFCA). They were located under the ligatures on the periosteal surface of the femoral neck, and blood circulation from those branches was physically blocked. The lateral epiphyseal arteries and the extracapsular arterial ring were attached to peri-osseous tissues that surround the femoral head and neck. The arterial network associated with the ligamentum teres was surgically detached, but no major arteries linked to the femoral neck were identified.

### Angiogram of a Pig Lower Body

**Figure 2:**
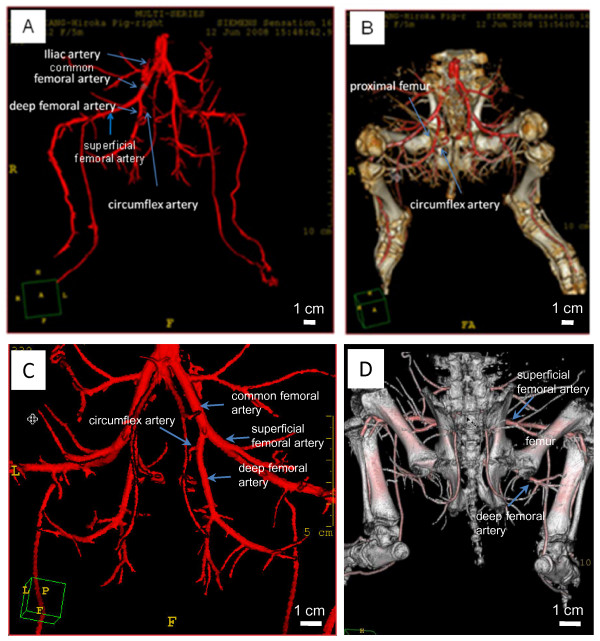
**CT images of the lower pig body**. (A) Anterior view showing the major blood vessels including the iliac, the common femoral, the superficial femoral, the deep femoral and the circumflex arteries. (B) Anterior view showing the blood vessels and skeletal structures. (C) Posterior view showing the major blood vessels including the iliac, the common femoral, the superficial femoral, the deep femoral and the circumflex arteries. (D) Posterior view showing the blood vessels and skeletal structures.

**Figure 3:**
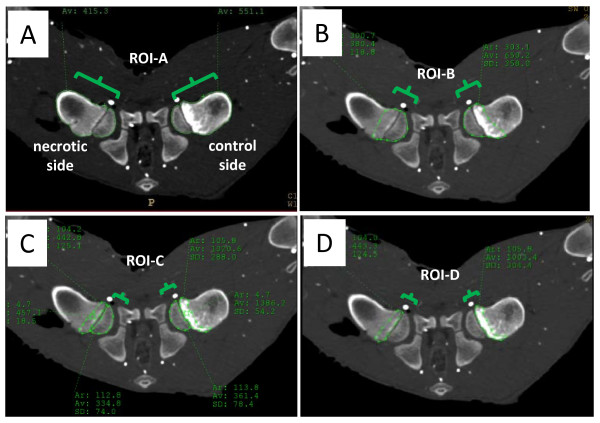
**Cross-sectional CT image highlighting four regions of interest**. (A) ROI-A: femoral head and neck. (B) ROI-B: whole femoral head. (C) ROI-C: proximal femoral head. (D) ROI-D: base of the femoral head.

CT images illustrated the distribution of major blood vessels in the lower body including the iliac, the common and superficial femoral, the deep femoral, and the circumflex arteries. Figure 2 illustrates the blood vessels with and without skeletal structures. The cross-sectional CT images were used to reconstruct the three-dimensional geometries, which highlighted the differences in the vascular networks between the control and the necrotic samples (Figure 3).

### Estimation of Blood Circulation in the Femoral Head

**Figure 4:**
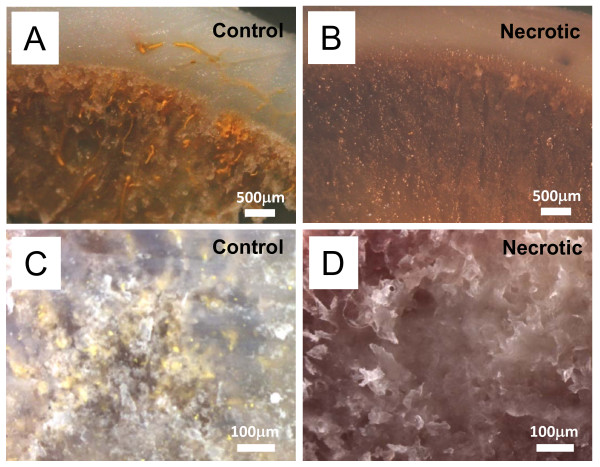
**Microfil perfused cross-sectional images**. (A) Peripheral image of the control femoral head. Bar = 500 μm. (B) Peripheral image of the necrotic femoral head. Bar = 500 μm. (C) Internal image of the control femoral head. Bar = 100 μm. (D) Internal image of the necrotic femoral head. Bar = 100 μm.

By focusing on the epiphysis and the metaphysis of the femoral head, the microfil casted blood vessels were examined. In the control section a network of yellow-stained (color of microfil used) vessels was visible in the peripheral and the center of the sections (Figure 4A and 4C). However, microfil staining was completely absent in the proximal femur that was tightly tied with the two ligatures (Figure 4B and 4D).

**Figure 5:**
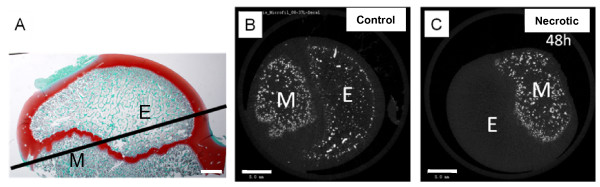
**Optical and CT images of the proximal femur**. The labels are E: epiphysis, and M: metaphysis. Bar = 5 mm. (A) Optical sagittal section with the transverse plane including the epiphysis and the metaphysis. (B) CT image of the control femoral head. (C) CT image of the necrotic femoral head.

After decalcification CT images on the plane including the epiphysis and the metaphysis confirmed that blood circulation was absent in the necrotic section but present in the control section (Figure 5). The reduction in image density is summarized in Table 1. In the base of the femoral head, for instance, the maximum reduction in density of 55.8% was detected in the necrotic femur.

**Table 1:** Comparison of image density in the proximal femur

ROI	region	control (HU)	necrotic (HU)	reduction (%)
A	head and neck	551	415	24.6

B	whole head	650	380	41.5

C	proximal head	361	335	7.4

D	head base	1003	443	55.8

## Discussion

One of the major causes of induction of avascular osteonecrosis is a lack of blood supply to the femoral head. Using the pig avascular osteonecrosis model we investigated the routes of blood circulation to the femoral head. Optical and CT imaging revealed that the effects of two ligatures in the femoral neck were responsible for reduction in blood supply to the femoral head. Collateral circulation from the metaphysis to the epiphysis through transphyseal vessels was not observed. That is, a complete lack of microfil perfusion was recorded. The results obtained from the microfil casted samples described herein are consistent with those derived from an earlier, microsphere-based detection technique [32].

Among several routes, MFCA contributed supplying blood to the femoral head through the extracapsular arterial ring. MFCA normally arises from the posteromedial aspect of the deep femoral artery, but it occasionally arises from the common femoral artery. It has multiple branches that enter the capsule of the hip joint along the femoral neck towards the femoral head. Besides MFCA, LFCA also supplies blood to the femoral head through the extracapsular arterial ring.

The current pig osteonecrosis study revealed a strong similarity in vascular patterning to the vascular anatomy of the human femora head [26,27]. In humans, vessels such as the extracapsular arterial ring, the artery of ligamentum teres, and the ascending femoral neck vessels are present in the proximal femur [33-35]. In the study, we identified the same vessels in the pig model with variations only in exact locations of arterial branches. Thus, the current pig model can be useful to evaluate blood circulation and pathogenetic alterations of avascular osteonecrosis of human femoral head. We also note that blood circulation may differ depending on age of animal, individual differences between animals, and differences between species, as well as variations in surgical procedures. It has been reported that the vessels of the ligamentum teres in the human femoral head do not contribute to the circulation of the femoral head during the early stage of growth period (from birth to the age of 4). Those vessels do, however, contribute after the age of 8 or 9 [26]. Since the 5-week old pigs used in this study are in the early stage of growth period, the findings obtained from the current pig model are referable to vascular anatomy of the human femoral head during growth.

Because of its relevance to clinical problems, the current animal model is suitable to understanding of pathological mechanisms for femoral head osteonecrosis. Perthes' disease, for instance, is avascular necrosis of the femoral head in a growing child [36]. It is known that blood circulation to the femoral head is reduced, but the exact cause of the reduction remains unclear. For fracture-linked osteonecrosis of the femoral head, our model may provide a useful tool for evaluating the effects of vascular damage near the femoral neck fracture site on progression of osteonecrosis of the femoral head [37].

In summary, the current study with avascular osteonecrosis of the pig femoral head demonstrates a critical role of blood vessels around the femoral neck. Augmenting blood flow around the femoral neck region could be a therapeutic target for an enhancement of blood supply. For instance, application of shock waves or pulsed electromagnetic fields might be useful to stimulate blood circulation.

## Conclusion

Optical and CT imaging revealed that the ligatures tightly tied around the femoral neck were responsible for the blockage of blood circulation and induction of osteonecrosis in the femoral head. Blood circulation to the femoral head is contributed by MFCA, LFCA, the extracapsular arterial ring, and the lateral epiphyseal arteries. Thus, enhancement of blood flow in these arteries may represent potential therapeutic strategy for avascular osteonecrosis.

## Competing interests

The authors declare that they have no competing interests.

## Authors' contributions

PZ and HK performed the animal experiments and drafted the manuscript. YL conducted CT imaging. HY designed the project and edited the manuscript. All authors read and approved the final manuscript.
